# Microglia P2X4R-BDNF signalling contributes to central sensitization in a recurrent nitroglycerin-induced chronic migraine model

**DOI:** 10.1186/s10194-019-1070-4

**Published:** 2020-01-14

**Authors:** Ting Long, Wei He, Qi Pan, Shanshan Zhang, Dunke Zhang, Guangcheng Qin, Lixue Chen, Jiying Zhou

**Affiliations:** 1 Department of Neurology, The Affiliated Hospital of Southwest Medical University, Luzhou, China; 2 Department of Neurology, The First Affiliated Hospital of Chongqing Medical University, 1st Youyi Road, Yuzhong District, Chongqing, 400016 China; 3 Laboratory Research Center, The First Affiliated Hospital of Chongqing Medical University, Chongqing, China

**Keywords:** Chronic migraine, Central sensitization, Microglia, P2X4R, BDNF

## Abstract

**Background:**

According to our previous study, microglia P2X4 receptors (P2X4Rs) play a pivotal role in the central sensitization of chronic migraine (CM). However, the molecular mechanism that underlies the crosstalk between microglia P2X4Rs and neurons of the trigeminal nucleus caudalis (TNC) is not fully understood. Therefore, the aim of this study is to examine the exact P2X4Rs signalling pathway in the development of central sensitization in a CM animal model.

**Methods:**

We used an animal model with recurrent intermittent administration of nitroglycerin (NTG), which closely mimics CM. NTG-induced basal mechanical and thermal hypersensitivity were evaluated using a von Frey filament test and an increasing-temperature hot plate apparatus (IITC). We detected P2X4Rs, brain-derived neurotrophic factor (BDNF) and phosphorylated p38 mitogen-activated protein kinase (p-p38-MAPK) expression profiles in the TNC. We investigated the effects of a P2X4R inhibitor (5-BDBD) and an agonist (IVM) on NTG-induced hyperalgesia and neurochemical changes as well as on the expression of p-p38-MAPK and BDNF. We also detected the effects of a tropomyosin-related kinase B (TrkB) inhibitor (ANA-12) on the CM animal model in vivo. Then, we evaluated the effect of 5-BDBD and SB203580 (a p38-MAPK inhibitors) on the release and synthesis of BDNF in BV2 microglia cells treated with 50 μM adenosine triphosphate (ATP).

**Results:**

Chronic intermittent administration of NTG resulted in chronic mechanical and thermal hyperalgesia, accompanied by the upregulation of P2X4Rs and BDNF expression. 5-BDBD or ANA-12 prevented hyperalgesia induced by NTG, which was associated with a significant inhibition of the NTG-induced increase in phosphorylated extracellular regulated protein kinases (p-ERK) and calcitonin gene related peptide (CGRP) release in the TNC. Repeated administration of IVM produced sustained hyperalgesia and significantly increased the levels of p-ERK and CGRP release in the TNC. Activating P2X4Rs with ATP triggered BDNF release and increased BDNF synthesis in BV2 microglia, and these results were then reduced by 5-BDBD or SB203580.

**Conclusions:**

Our results indicated that the P2X4R contributes to the central sensitization of CM by releasing BDNF and promoting TNC neuronal hyper-excitability. Blocking microglia P2X4R-BDNF signalling may have an effect on the prevention of migraine chronification.

## Introduction

Migraine is a complex and severe neurological disorder characterized by repeated attacks. Compared with episodic migraine, chronic migraine has a greater financial burden on global economies [1]. Although chronic migraine typically progresses from episodic migraine, the mechanisms underlying this progression are not understood. Some clinicians have suggested that a high frequency of headaches is an important risk factor for progression [2]. Emerging evidence supports that central sensitization is related to the pathophysiological mechanism of chronic migraine [3].

Central sensitization refers to a condition where central neurons in the trigeminal nociceptive pathway, principally the trigeminal nucleus caudalis (TNC), exhibit increased excitability. Clinically, central sensitization is manifested as cutaneous allodynia and an exaggerated range of pain responses, such as in the forearms and trunk. Recent evidence suggests that microglia surrounding TNC neurons directly or indirectly influence the establishment of central sensitization. Previous results from our team have indicated that microglial activation was correlated with NTG-induced hypersensitivity in C57BL/6 mice and also had an effect on central sensitization induced by chronic intermittent nitroglycerin (NTG) [4]. However, the molecular mechanism that underlies the crosstalk between microglia and neurons of the TNC needs further study.

P2X4 receptors (P2X4Rs) belong to the family of purinergic P2 receptors, which have been extensively studied in neuropathic pain [5]. The first observation of P2X4Rs in neuropathic pain was in 2003 [6]. The results indicated that after nerve injury, the expression of P2X4Rs in the spinal cord was up-regulated exclusively in microglia, not in neurons or astrocytes. In addition, blocking P2X4Rs could suppress tactile allodynia induced by nerve injury. After this discovery, a growing body of evidence from diverse animal models of neuropathic pain indicated that microglial P2X4Rs were an important player in the mechanism of neuropathic pain. However, the exact roles of activated microglia and P2X4Rs are not fully understood in migraine. In our previous studies, we found that the expression of P2X4Rs was increased in the TNC after repeated NTG stimulation [4]. P2X4Rs were associated with NTG-induced hyperalgesia and the changes in neurochemical signs accompanying migraine in the TNC, such as the signalling of c-Fos and calcitonin gene related peptide (CGRP). However, a key unresolved question is how microglial P2X4Rs affect TNC neuronal excitability. The exact downstream pathways of P2X4Rs and the key molecule mediating this microglia–neuron signalling are not clear.

Microglia are considered innate immune cells in the central nervous system. When microglia are activated, a variety of neuroexcitatory substances, including reactive oxygen species (ROS), and inflammatory cytokines are produced and released. Brain-derived neurotrophic factor (BDNF) is a pivotal chemical mediator that maintains information transmission between microglia and neurons. An increasing number of studies have suggested that BDNF is expressed in the trigeminovascular system and has a role in migraine pathophysiology [7]. Pre-clinical research on neuropathic pain has demonstrated that microglial P2X4Rs stimulated the synthesis and release of BDNF and that BDNF could alter dorsal horn neuronal excitability [8]. To our knowledge, no study has examined the exact mechanisms involved in the role of microglia P2X4Rs in migraine.

The aim of this research was to investigate whether microglia P2X4Rs contribute to repeated NTG-induced central sensitization by regulating the expression of BDNF in the TNC. Here, we used intermittent NTG injection to build a chronic migraine animal model. The migraine-related behavioural and neurochemical changes were investigated in the TNC of the model mice.

## Materials and methods

### Animals

Male C57BL/6 mice weighing 18–20 g and between 8 and 10 weeks old were obtained from the Experimental Animal Center of Chongqing Medical University (Chongqing, China). The mice were kept in standard laboratory conditions with a 12–12 light-dark cycle and enough food and water. All procedures followed the National Institutes of Health Guidelines for the Care and Use of Laboratory Animals. After a week of adaptation, the animals were randomly assigned to experimental groups using the ‘rand()’ function of Excel software.

### Animal modelling and experimental design

A stock solution of 5.0 mg/ml NTG (Beijing Regent, China), dissolved in 30% alcohol, 30% propylene glycol and water, was used to construct the animal model. NTG was diluted in 0.9% saline at 1 mg/ml prior to the start of the experiment. The vehicle control was 0.9% saline. There was no significant difference in mechanical thresholds between the 0.9% saline solution and the solution in which NTG was dissolved (6% propylene glycol, 6% alcohol, 0.9% saline) [9]. The animals received intraperitoneal (i.p.) injections of 10 mg/kg NTG or vehicle every other day for 9 days (i.e., days 3, 5, 7, 9 and 11).

#### Experiment 1

To understand the effect of the P2X4R/BDNF pathway on NTG-induced hyperalgesia, we treated the animals with the P2X4R-selective antagonist 5-BDBD (Sigma-Aldrich, USA) and the inhibitor of TrkB receptors ANA-12 (Sigma-Aldrich, USA) daily for 11 days; on days 3, 5, 7, 9, and 11, the mice were injected with NTG as described above. 5-BDBD (28 mg/kg, i.p.) and ANA-12 (1 mg/kg, i.p.) were administered immediately prior to the administration of NTG [10, 11].

#### Experiment 2

The P2X4R-selective agonist IVM (Sigma-Aldrich, American) was intraperitoneally injected every other day for 9 days (i.e., days 1, 3, 5, 7, and 9), similar to NTG administration. IVM was administered in 2 doses (0.1 mg/kg or 1 mg/kg) [12]. All these drugs were dissolved in DMSO solution.

To understand the temporal expression profiles of the P2X4R, p38 mitogen-activated protein kinase (p38-MAPK), p-p38-MAPK and BDNF in the animal model, samples of the TNC (running caudally approximately 2 mm from the obex) for Western blot were collected 2 h after different NTG injections (i.e., days 3, 7, 9 and 11). For the double immunolabelling of P2X4Rs and BDNF, the TNC was sampled 2 h after the last NTG injection (i.e., day 11). To examine the effects of 5-BDBD, ANA-12, and IVM on migraine-related biochemical changes, samples were collected 2 h after the last NTG injection (i.e., day 11) for CGRP (Immunofluorescence). Due to the short half-life of phosphorylated extracellular regulated protein kinases (p-ERK), the TNC was sampled 10 min after the last administration of NTG for Western blotting.

### Behavioural tests

**Fig. 1:**
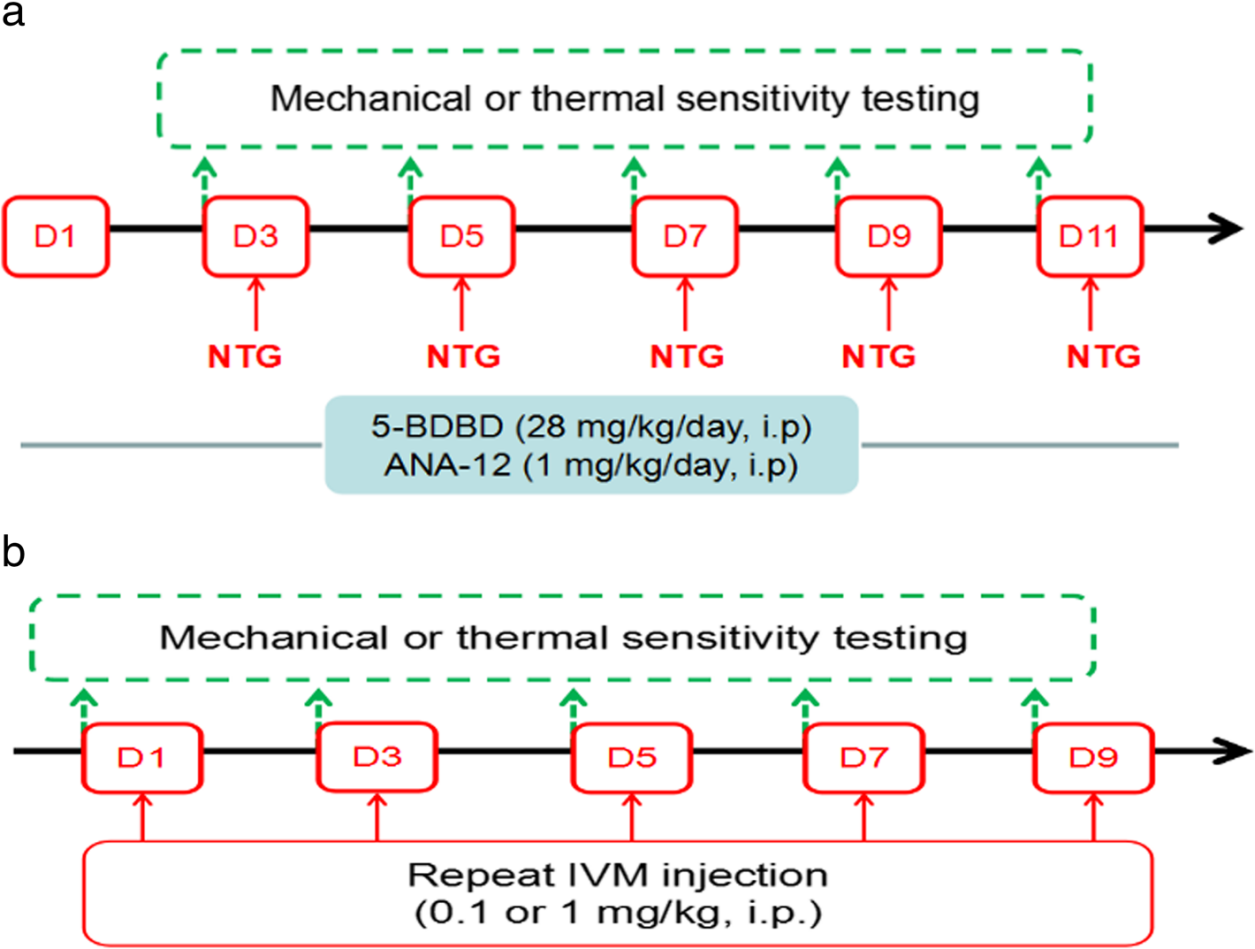
Experimental flow chart. **a**. Experimental design for studying the effect of the P2X4R-selective antagonist 5-BDBD and the inhibitor of TrkB receptors ANA-12 on NTG-induced hyperalgesia. NTG: nitroglycerin. D: day. **b**. Experimental design for studying the effect of P2X4R-selective agonist IVM on the effect of mechanical and thermal sensitivity

Behaviour testing occurred under light conditions between 09:00 and 15:00 by an experimenter blinded to the treatment. The treatment of the mice on the testing days was as follows: mice were habituated to the test room, followed by a baseline measurement of mechanical or thermal sensitivity 15–20 min later and administration of 5-BDBD, ANA-12 or vehicle; then, an injection of NTG/saline was given. For the IVM chronic experiments, IVM was administered following sensory sensitivity testing. This procedure was repeated every other day for 9 days (see Fig. 1a, b).

The paw withdrawal threshold (PWT, g), as an indicator of mechanical sensitivity, was assessed using an Electro-vonFrey Aesthesiometer with a rigid plastic tip (Woodland Hills, CA, USA). The animals were habituated in a transparent Plexiglas box with a raised mesh grid bottom for 15–20 min. The tip of the device was then applied with increasing force to the middle of the plantar surface of the paw (avoiding the footpads) from below. The positive withdrawal response was the rapid retraction of the hind paw away from the rigid tip. The PWT were measured at least three times in each animal, with an interval of at least 3 min. The average of the three measurements was considered the PWT [13].

The paw withdrawal latency (PWL, s) to noxious heat, as an indicator of thermal sensitivity, was assessed using an increasing-temperature hot plate apparatus (IITC). The mice were placed within a transparent Plexiglas box and allowed to habituate to the environment for 20–30 min. An infrared beam with an adjustable intensity beneath the glass platform was applied at the plantar surface of the hind paw. The noxious heat stimulus was set to 50% intensity, and the cut-off time was 10 s to avoid damage to the hind paw. At least three individual measures were taken in each animal with at least a 5 min interval. The average of the three measurements was considered the PWL [14].

### Western blotting

The TNC samples were homogenized in RIPA lysis buffer for 1 h. The protein concentration was determined using a BCA protein assay kit (Beyotime, China). The proteins were separated on an SDS-PAGE gel, electrophoresed, and transferred to PDVF membranes. Following transfer, the membranes were blocked for 2 h at room temperature in TBST containing 5% non-fat milk and then incubated overnight at 4 °C with the appropriate antibodies diluted in TBST: rabbit anti-P2X4R (1/500, Abcam), rabbit anti-EKR1/2 (1:1000, CST), mouse anti-BDNF (1:1000, Abcam) and rabbit anti-p38-MAPK and p-p38-MAPK (1:1000, CST). The next day, after three washes in TBST, the membranes were incubated with specific HRP-conjugated secondary antibodies for 2 h at room temperature and revealed with an ECL detection kit (Advansta). β-Actin (1:9000, Proteintech, China) was used as a control to normalize protein levels.

### Immunofluorescence staining and counting

The mice were anaesthetized and perfused transcardially with 4% paraformaldehyde (PFA). Brain stems were collected and post-fixed in 4% PFA for 24 h. The medullary segment containing the TNC between + 1 and − 3 mm from the obex was removed and subsequently transferred in solutions of sucrose at 20% and 30% concentrations for 72 h. All samples were cut transversely at 10 μm on a freezing sliding microtome (Leica).

The sections were washed three times with PBS and permeabilized with 0.3% Triton X-100 (Beyotime, China) in 0.1 M PBS at 37 °C for 10 min and then incubated with 10% normal goat serum (Boster, China) at 37 °C for 30 min. For the identification of TNC P2X4R, BDNF and CGRP, the sections were incubated with goat anti-rabbit P2X4R polyclonal antibody (1:200, Abcam), goat anti-mouse BDNF polyclonal antibody (1:100, Santa Cruz), or goat anti-mouse CGRP antibody (1:100, Santa Cruz) overnight at 4 °C. The next day, after three washes in PBS, the sections were incubated for 2 h with secondary species-specific fluorophore-labelled secondary antibody (Abbkine). Nuclei were counterstained with 4′,6-diamidino-2-phenylindole (DAPI) at 37 °C for 5 min. The sections were viewed under a confocal laser scanning fluorescence microscope (ZEISS, Germany).

The TNC region was determined based on the morphological appearance under light microscopy using the Mouse Brain Atlas as a reference [15]. Images were taken under the 10× objective of a ZEISS confocal fluorescence microscope (Germany). The area covered by CGRP-immunoreactive fibres was determined using Image Pro Plus 6.2 image analysis software (Media Cybernetics), as suggested by previous reports. The TNC region, including laminae I-IV, was determined manually as an area of interest. All evaluations were performed in a blinded fashion on 6–8 TNC sections per animal. In addition, both sides of each section were analysed, and the mean value of the two sides was subsequently calculated.

### BV2 microglial cell cultures and treatments

BV2 microglial cells (kindly provided by the Neurology Laboratory, the First Affiliated Hospital of Chongqing Medical University) were cultured in DMEM-F12 supplemented with 10% FBS, 100 U/ml penicillin, and 100 U/ml streptomycin at 37 °C in a humidified environment containing 5% CO2. Cell cultures were stimulated with ATP at a 50 μM concentration for distinct time-points, i.e., 0 h, 30 min, 1 h, 2 h and 3 h. This concentration of ATP has been verified to activate the P2X4Rs but not the P2X7Rs [6]. To examine the role of P2X4Rs and p38-MAPK in the ATP-evoked release and synthesis of BDNF, beginning 15 min before ATP stimulation, the cells were treated with 5-BDBD (20 μM, Sigma), an antagonist of the P2X4Rs, and SB203580 (10 μM, MCE), a p38-MAPK inhibitor. ATP was applied for 2 h [10, 16].

### Enzyme-linked immunosorbent assay (ELISA)

The levels of BDNF secreted from BV2 cells into the supernatant were measured by using a BDNF-specific ELISA kit (no. JYM0083Mo, ELISA LAB Co., China). Briefly, after the application of ATP and other drugs, 200 μl of the supernatant sample was collected. According to the manufacturer’s instructions, the samples were added to the appropriate microelisa stripplate wells and combined with specific antibodies. Then, HRP-conjugated antibodies specific for BDNF were added to each well and incubated. Chromogen Solutions A and B were added for colouring. Stop solution was finally added to terminate the reaction. The optical density (OD) was measured spectrophotometrically at a wavelength of 450 nm.

### Immunocytochemistry staining

Immunocytochemistry staining was performed to detect BDNF protein in the cultured BV2 cells. Briefly, the cells plated on coverslips were fixed for 10 min with 4% paraformaldehyde in PBS at room temperature. After three washes in PBS, the samples were incubated for 10 min with PBS containing 0.3% Triton X-100 and then blocked with 10% FCS for 30 min at 37 °C. The samples were incubated overnight at 4 °C with primary antibodies [mice-anti-BDNF 1/100 (Abcam), goat-anti-iba1 1/400 (Abcam), or rabbit-anti-P2X4R 1/200, (Proteintech)]. After three washes in PBS, the cells were incubated for 1 h with secondary species-specific antibodies [donkey-anti rabbit Alexa555, donkey-anti mice Alexa555, or donkey-anti goat Alexa488 secondary antibody (Abcam)].

### Statistical analysis

Data are expressed as the mean ± SD. All statistical analyses were performed by GraphPad Prism version 6.0 (Graph Pad Software Inc., San Diego, CA). Behavioral results were analyzed using a two-way RM ANOVA, with drug and time as factors. In this case, all groups were compared to responses on day 1 or 3, and to the VEH-VEH group. Unless otherwise noted, all experiments were analyzed using one-way ANOVA followed by Tukey’s Multiple comparison test. A significance level of *p* < 0.05 was used.

## Results

### P2X4Rs are upregulated after recurrent NTG injection

**Fig. 2:**
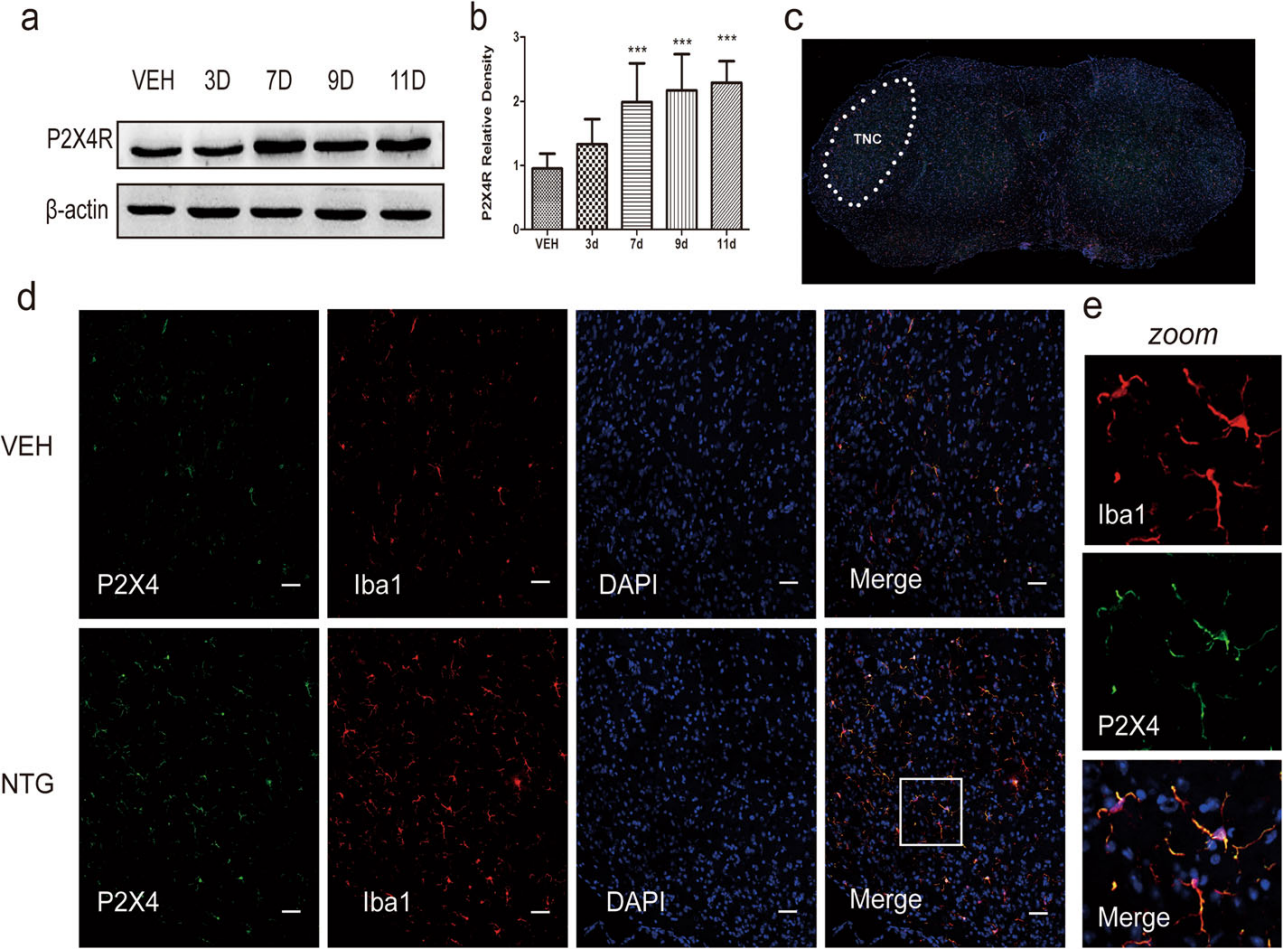
Expression profile of the P2X4R in the TNC after the recurrent intermittent administration of NTG. **a**, **b** Western blot assay for the expression profile of P2X4Rs in VEH and NTG mice on days 3, 7, 9, and 11. One-way ANOVA and Tukey’s multiple comparison test. *n* = 6/group, ***p < 0.001 versus VEH. **c** The white dotted line frame indicates the TNC regions. **d** Representative photographs of immunofluorescence staining for P2X4R (green) expression in microglia (Iba-1, red) in the TNC in the VEH and NTG groups on day 11. Scale bar = 50 μm. **e** The white box was amplified. Scale bar = 20 μm

To examine the temporal expression profile of the P2X4Rs following NTG, we detected the levels of P2X4Rs protein in the TNC. The Western blot results showed that P2X4Rs protein was significantly increased on day 7 in the NTG group compared with that in the VEH group. On the last day of NTG injection (i.e., day 11), the expression of P2X4Rs was still high (Fig. 2a, b). Furthermore, double immunofluorescence staining showed that P2X4Rs were mainly double-labelled with Iba1 (a microglia-specific marker) (Fig. 2d, e), indicating that the P2X4Rs were expressed in microglia, as shown in previous research [6].

### The P2X4Rs antagonist 5-BDBD attenuates NTG-induced hypersensitivity

**Fig. 3:**
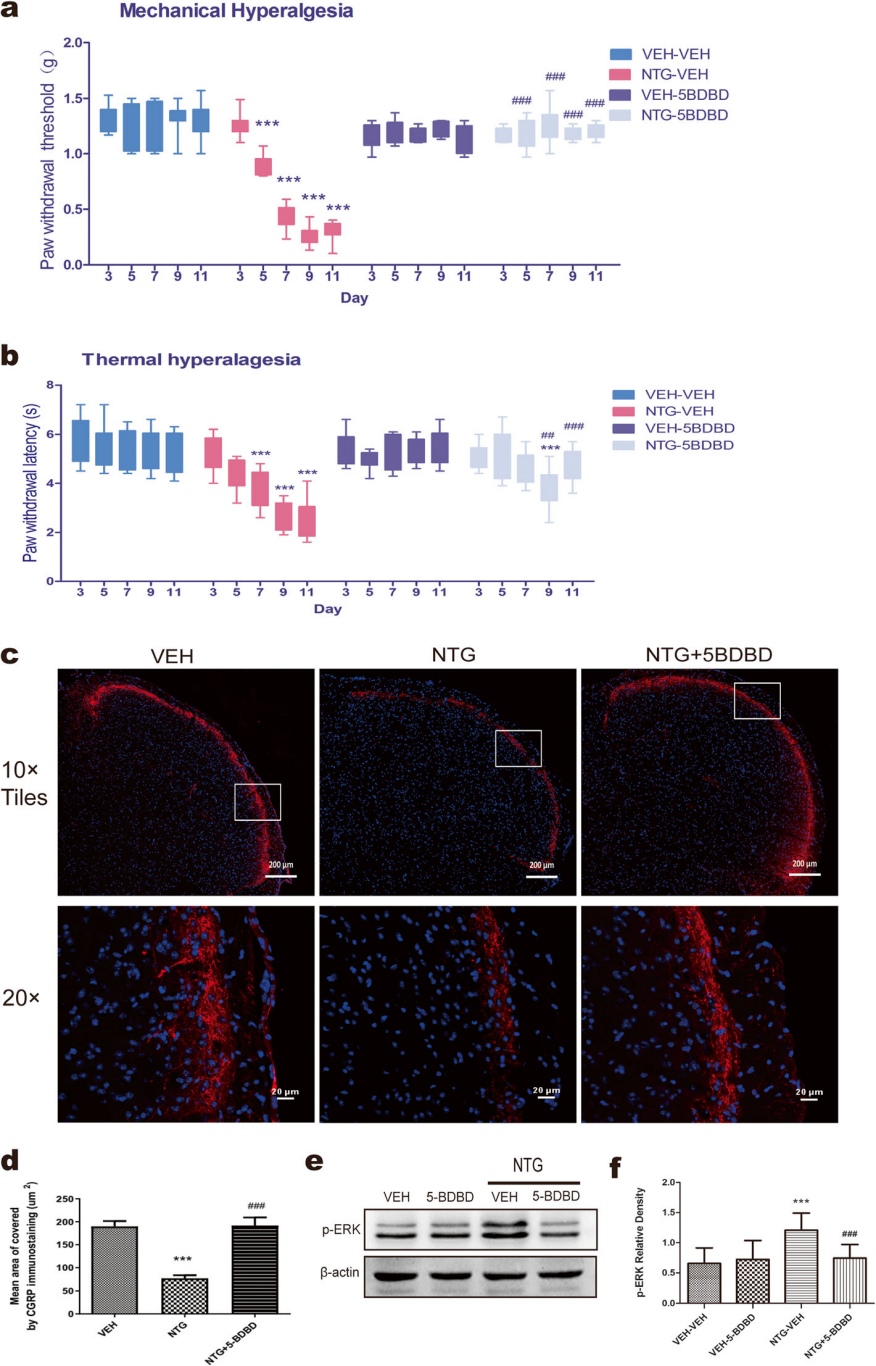
Effect of 5-BDBD on NTG-induced migraine-related behavioural and neurochemical changes. NTG (10 mg/kg, i.p.) or saline was injected every other day from day 3 to day 11. Mice were treated daily with vehicle (VEH) or 5-BDBD (28 mg/kg, i.p.) for 11 days. **a**, **b** Mechanical and thermal sensitivity were assessed in different groups. *p* < 0.01 for drug, time, and interaction. Two-way RM ANOVA and Bonferroni post hoc analysis. *n* = 10/group. ***p < 0.001 versus VEH-VEH group. ##*p* < 0.01, ###*p* < 0.001 versus NTG-VEH group. **c** Representative photos of CGRP-immunoreactive staining in the TNC of the VEH, NTG and NTG + 5-BDBD groups under a 10 × objective lens (the upper row, scale bar = 200 μm) or 20 × objective lens (the lower row, scale bar = 20 μm). The white box was enlarged. **d** Mean area covered by CGRP-immunoreactive fibres in different groups. One-way ANOVA and Tukey’s multiple comparison test. *n* = 8/group, ***p < 0.001 versus VEH, ###*p* < 0.001 versus NTG. **e**, **f** Representative Western blot results for p-ERK in different groups. One-way ANOVA and Tukey’s multiple comparison test. *n* = 8/group, ***p < 0.001 versus VEH-VEH, ###*p* < 0.001 versus NTG-VEH

To determine whether the change in the P2X4Rs was involved in the development and maintenance of pain hypersensitivity, 5-BDBD, a P2X4R-specific antagonist (28 mg/kg), was administered by intraperitoneal injection. In agreement with previous findings, repeated injection of NTG significantly reduced the paw withdrawal threshold (PWT) (Fig. 3a) and paw withdrawal latency (PWL) (Fig. 3b) on days 5, 7, 9, and 11 compared with VEH, which suggested the development of mechanical and thermal sensitivity hyperalgesia. However, following treatment with 5-BDBD for 11 days, there was a significant improvement in the PWT and PWL as well as attenuated mechanical and thermal hyperalgesia induced by NTG. Furthermore, in the mechanical sensitivity test, the effect of 5-BDBD began on day 5 and gradually continued throughout the 11 days; in the thermal sensitivity test, 5-BDBD played a therapeutic role on day 9. In the VEH-5BDBD group, 5-BDBD injection alone did not change the mechanical and thermal responses compared to VEH injection.

### Chronic 5-BDBD treatment attenuates the neurochemical signs accompanying migraine in the TNC

CGRP is considered a biomarker of migraine headaches, and it is mainly synthesized in the trigeminal ganglion (TG) and released in the TNC [17]. Compared with VEH administration, repeated administration of NTG decreased the area covered by CGRP immunostaining in the superficial lamina of the TNC, which suggested that NTG administration may cause significant release of CGRP from presynaptic afferent terminals (*p* < 0.001, Fig. 3c, d). However, chronic 5-BDBD treatment prevented the reduction in CGRP immunostaining induced by NTG in the NTG + 5-BDBD group (*p* < 0.01), which is related to the effect of 5-BDBD on NTG-induced hyperalgesia.

Mitogen-activated protein kinases (MAPKs), including p-ERK, have been suggested to play a key role in inducing and maintaining peripheral and central sensitisation, not only in neuropathic pain but also in migraine [18, 19]. Our results observed an increase in p-ERK levels in the TNC after recurrent NTG injection compared to that after VEH injection (p < 0.001, Fig. 3e, f). In addition to the analgesic effects of 5-BDBD, our results also showed that the expression of p-ERK was inhibited by 5-BDBD.

### Repeated administration of IVM produces sustained hyperalgesia

**Fig. 4:**
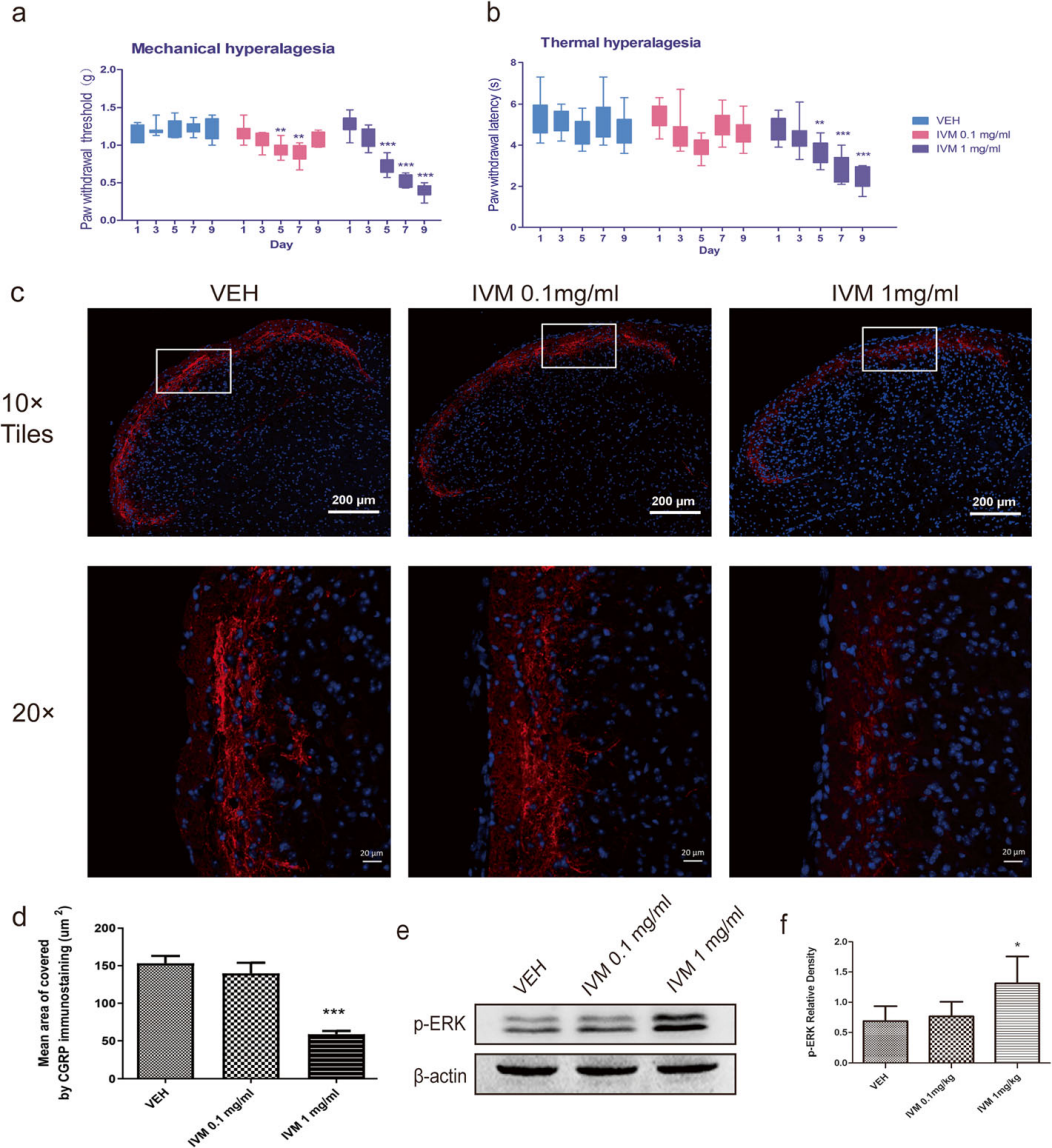
The effect of repeated IVM administration on migraine-related behaviours and neurochemical signalling in the TNC. Mice were treated with IVM every second day for 9 days with two doses (0.1 mg/ml or 1 mg/ml, i.p.). **a**, **b** Mechanical and thermal hyperalgesia were assessed in different groups. *p* < 0.01 for drug, time, and interaction. Two-way RM ANOVA and Bonferroni post hoc analysis. *n* = 9/group. **p < 0.01, ***p < 0.001 versus VEH group. **c** Representative photos of CGRP-immunoreactive staining in the TNC of different groups under a 10 × objective lens (upper row, scale bar = 200 μm) and 20 × objective lens (lower row, scale bar = 20 μm). The white box was enlarged. **d** Mean area covered by CGRP-immunoreactive fibres in different groups. One-way ANOVA. *n* = 8/group, ***p < 0.001 versus VEH. **e**, **f** Representative Western blot results for p-ERK in different groups. One-way ANOVA and Tukey’s multiple comparison test. *n* = 6/group, *p < 0.05 versus VEH

The repeated administration of systemic NTG was previously shown to produce thermal and mechanical hyperalgesia in mice, which produced a new reliable model of chronic migraine. We used the same protocol to test IVM to determine if the activation of P2X4Rs would produce a similar effect. In our research, we administered IVM every second day for 9 days in two doses (0.1 mg/ml or 1 mg/ml, i.p.). The PWT was significantly decreased in the 1 mg/kg IVM group, which was observed after 2 single IVM injections and did not return to normal on the final injection day, similar to the results observed in the NTG injections (Fig. 4a). However, low-dose IVM alone produced reversible paw mechanical hyperalgesia (Fig. 4a). In the thermal hyperalgesia test, the PWL to innocuous thermal stimuli was significantly lower in the high-dose IVM group than that in the VEH group or low-dose IVM group (Fig. 4b). In addition, there was no difference between the vehicle mice and the low-dose IVM mice. Together, these results indicate that P2X4Rs stimulation can produce mechanical and thermal hyperalgesia, which is very similar to that induced by NTG.

### IVM increases the CGRP release and the expression of p-ERK in the TNC

To determine whether the mechanical and thermal hyperalgesia induced by IVM was related to migraine, we tested the expression of CGRP and p-ERK in the TNC. With recurrent treatments, high-dose IVM induced a significant reduction in the area covered by CGRP immunostaining (Fig. 4c, d). Treatment with low-dose IVM did not show a significant effect compared with VEH treatment. In addition, we also found that compared to the VEH and low-dose IVM group, the high-IVM group demonstrated a much stronger change in the expression of p-ERK. No differences were observed between the VEH group and the low-dose IVM group (Fig. 4e, f). Altogether, these results show that chronic IVM stimulation is correlated with increased CGRP release from the TNC and p-ERK expression, which supports the notion that the sustained activation of P2X4Rs was related to repeat NTG-induced central sensitization.

### ATP triggers BDNF release and increases BDNF synthesis in BV2 microglia culture

**Fig. 5:**
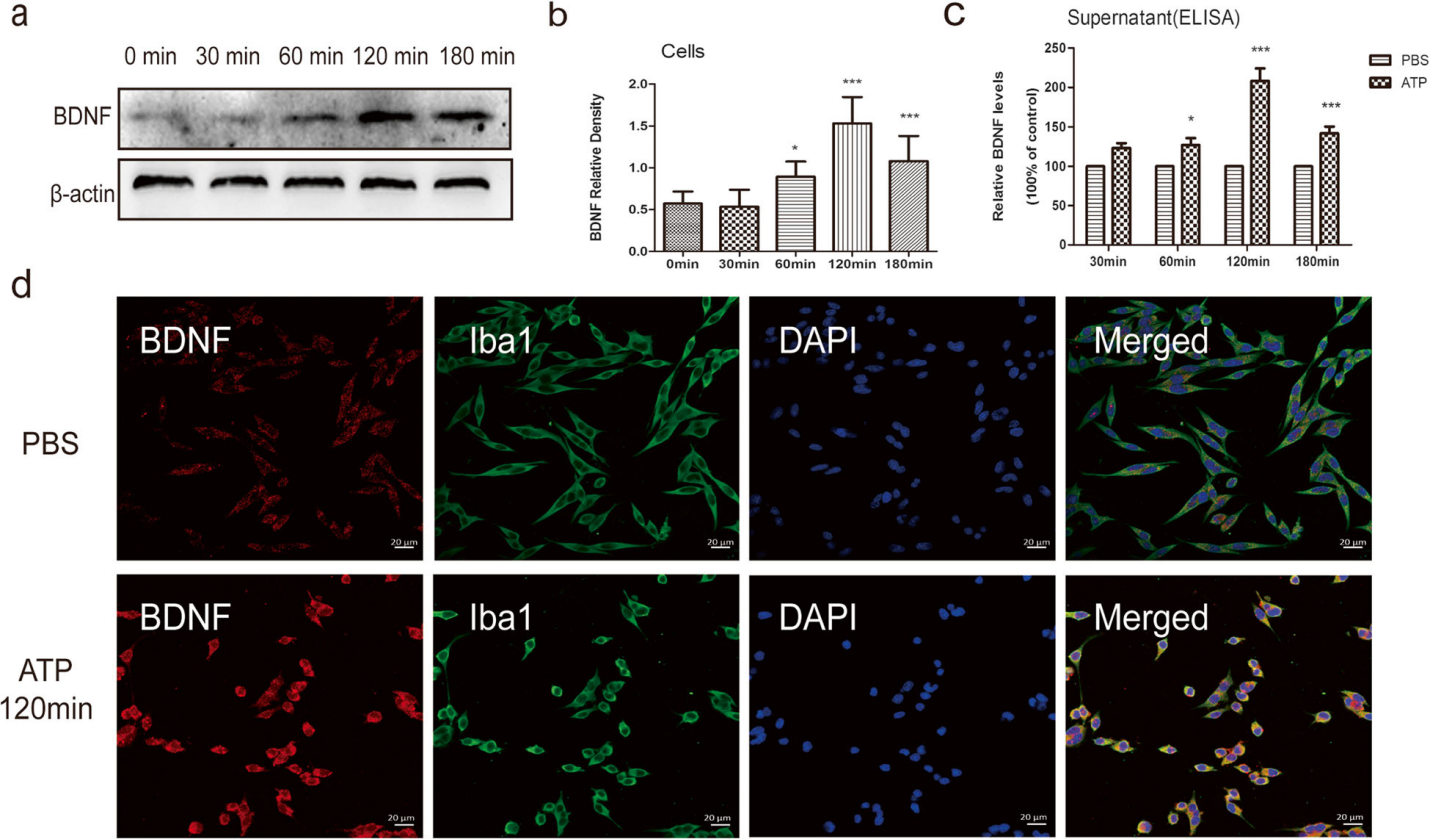
ATP enhanced BDNF synthesis and release in BV2 microglia cells. BV2 cells were treated with 50 μM ATP to activate P2X4Rs. At different time points, supernatants and cell lysates were collected. **a** Representative Western blot results of intracellular BDNF in BV2 cells. **b** The histogram presents the quantitative analysis of BDNF protein. *n* = 6/group, one-way ANOVA and Tukey’s multiple comparison test. *p < 0.05, ***p < 0.001 versus PBS. **c** Concentration of BDNF in the supernatants incubated with ATP at different time points. *n* = 8/group, T test. *p < 0.05, ***p < 0.001 versus PBS. **d** Immunofluorescence staining for BDNF (red) and Iba1 (green) at 120 min after PBS or ATP stimulation. Scale bar = 20 μm

It has been demonstrated that after peripheral nerve injury, the expression of microglia P2X4Rs and BDNF both increase in the spinal dorsal horn ipsilateral to the nerve injury, and these increases are in parallel with pain hypersensitivity [8]. To determine whether P2X4Rs stimulation causes the release of BDNF from microglia, we studied BV2 cells in vivo. The cells were treated with 50 μM ATP to activate the P2X4Rs but not the P2X7Rs at different time points. We first characterized the time course of BDNF protein synthesis in cell lysates after ATP stimulation by Western blot analysis. We found that the synthesis of BDNF increased starting at 60 min, peaked at 120 min after adding ATP, and maintained a high level until the last examination min after ATP stimulation, which was 180 min (Fig. 5a, b). In addition, we also measured BDNF secreted from BV2 cells into the supernatant. We found that the level of BDNF in the supernatant significantly increased at 60 min and peaked at 120 min, which was consistent with the results of Western blot (Fig. 5c). Immunofluorescence staining was measured for BDNF (red) and Iba1 (green) 120 min after PBS or ATP stimulation. Cellular localization of BDNF and Iba1 was clearly identified in BV2 microglia cells. After ATP treatment, the activation of BV2 microglial cells was detected, which was indicated by increased Iba1 immunoreactivity and changes in cell morphology (from shuttle to circle). In addition, slightly weak immunoreactivity of BDNF was observed in the PBS group, and strong immunostaining was observed 120 min after ATP exposure (Fig. 5d). These results indicated that stimulating P2X4Rs increased the expression of BDNF in microglia.

### Blocking P2X4Rs attenuates ATP-evoked secretion and the accumulation of BDNF

**Fig. 6:**
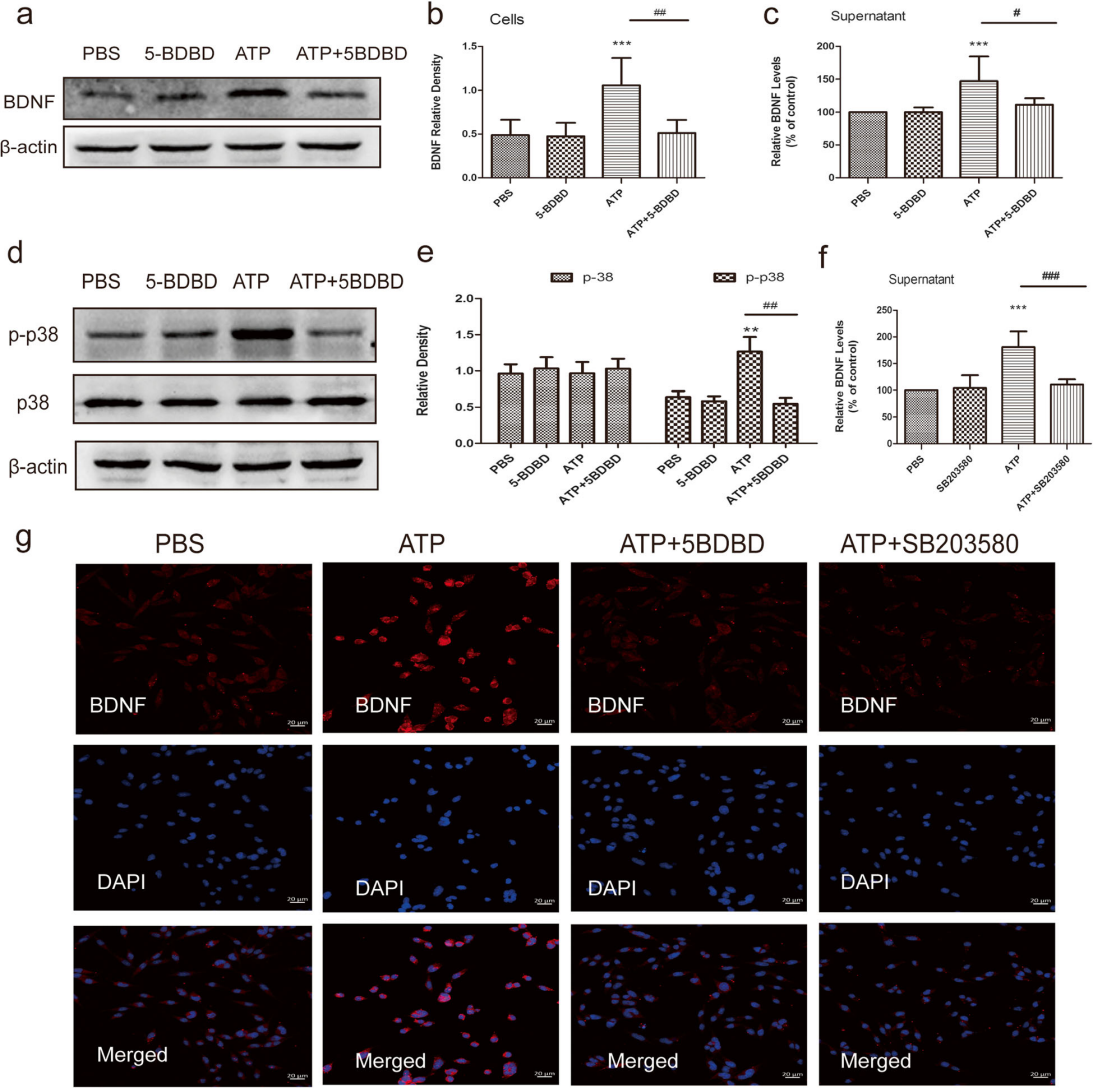
Inhibition of P2X4Rs attenuates ATP-evoked release and synthesis of BDNF, which depends on p38-MAPK. **a** Representative Western blot results of intracellular BDNF in cells pre-incubated with 5-BDBD (20 μM). **b** The histogram presents the quantitative analysis of BDNF protein. *n* = 6/group, One-way ANOVA, ***p < 0.001 versus PBS, ##*p* < 0.01 versus ATP. **c** Levels of BDNF in the supernatants of BV2 cells in different groups 120 min after drug treatment. *n* = 8/group, ***p < 0.001 versus PBS, #*p* < 0.05 versus NTG. **d** Effect of treatment with 5-BDBD on p38MAPK expression after ATP stimulation. Representative Western blot results of p38/p-p38MAPK in BV2 cells. **e** The histogram presents the quantitative analysis of p38/p-p38MAPK protein. *n* = 6/group, **p < 0.01 versus PBS, ##*p* < 0.01 versus ATP. **f** Effect of treatment with SB203580 on BDNF release after ATP stimulation. The histogram presents the levels of BDNF in the supernatants of BV2 cells. One-way ANOVA, ***p < 0.001 versus PBS, ###*p* < 0.001 versus ATP. **g** Immunofluorescence staining for BDNF (red) in BV2 cells at 120 min after drug treatments. Scale bar = 20 μm

5-BDBD is known to block P2X4Rs and reverses peripheral nerve injury-induced pain hypersensitivity. We found that with the pre-incubation of 5-BDBD (20 μM), ATP had no effect on the level of BDNF in the cell lysates or supernatant at 120 min time points (Fig. 6a-c). Moreover, 5-BDBD treatment alone did not affect the release and synthesis of BDNF. Immunofluorescence was further performed to confirm the ATP-induced upregulation of BDNF expression, which was greatly suppressed by pretreatment with 5-BDBD (Fig. 6g).

### Treatment with SB203580 suppresses ATP-evoked BDNF release

We next investigated the intracellular signalling pathways related to the release and synthesis of BDNF. p38-MAPK, a member of the MAPK family, is implicated in pain hypersensitivity after peripheral nerve injury [20]. Therefore, we detected the effect of p38-MAPK on P2X4R-stimulated release or the synthesis of BDNF in microglia. We found a significant increase in phospho-p38MAPK (p-p38-MAPK) expression and no change in the total expression of p38-MAPK protein after ATP exposure (Fig. 6d, e). However, the increase in p-p38-MAPK was prevented by 5-BDBD. The p38-MAPK inhibitor SB203580 (10 μM) prevented the release of BDNF 120 min after the addition of ATP. Moreover, there was no significant difference in BDNF release between the PBS and SB203580 groups (Fig. 6f). Immunofluorescence further showed that pretreatment with SB203580 suppressed the ATP-induced upregulation of BDNF immunoreactivity in the cells (Fig. 6g). Therefore, we conclude that p38-MAPK is the signalling pathway for P2X4Rs that triggers BDNF release and expression.

### P-p38-MAPK and BDNF increase following recurrent NTG injection, and this is reversed by a P2X4R inhibitor

**Fig. 7:**
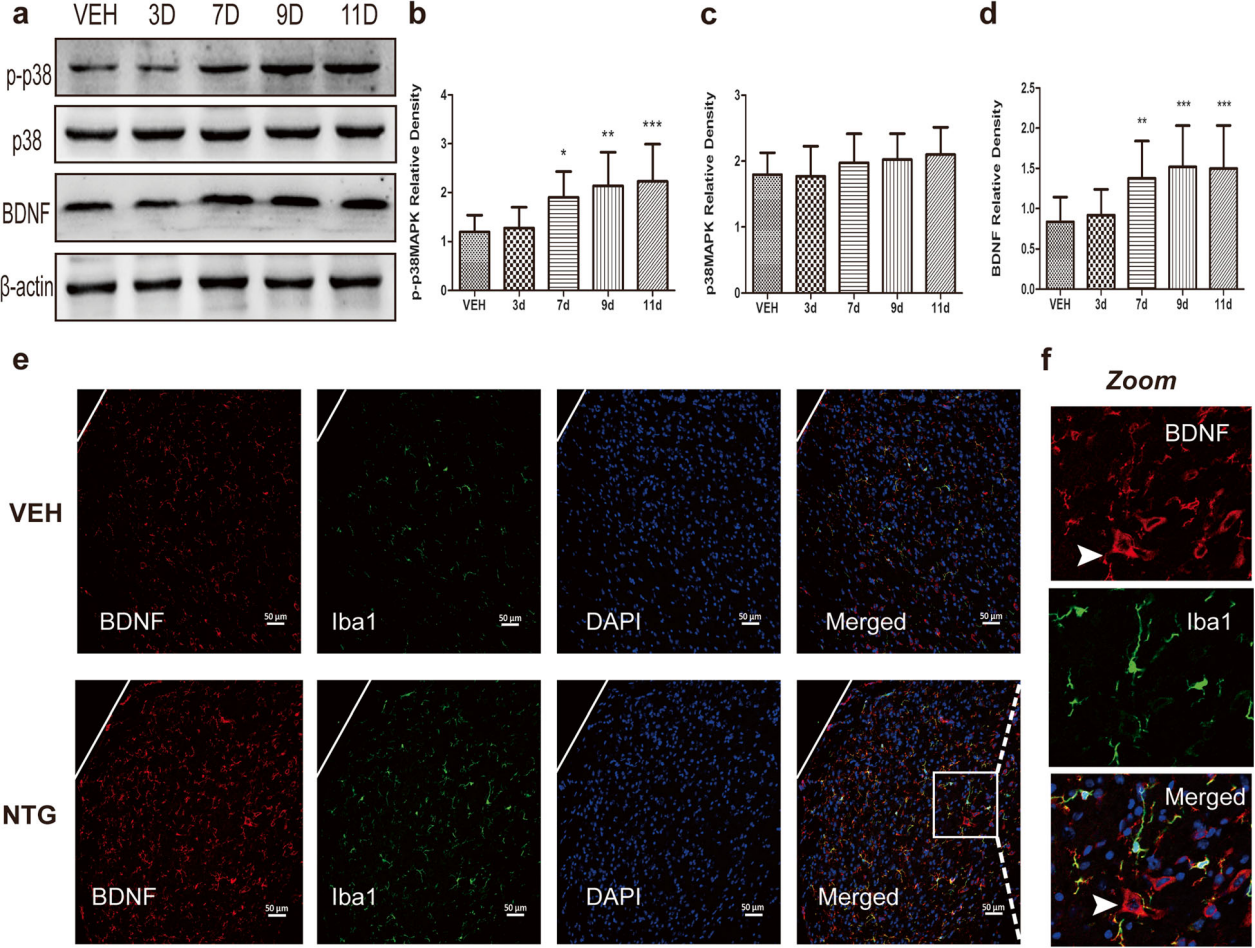
Effect of NTG on p38, p-p38MAPK and BDNF levels in the TNC. **a** Representative images of Western blot results showing the p38, p-p38MAPK and BDNF protein levels from 3 d to 11 d during the NTG injection. **b**-**d** The histogram presents the quantitative analysis of p-p38 (**b**), p38MAPK (**c**) and BDNF (**d**) in the TNC. Data are presented as the mean ± SD. One-way ANOVA and Tukey’s multiple comparison test. *p < 0.05, **p < 0.01, ***p < 0.001 versus VEH. **e** Representative photographs of immunofluorescence staining for BDNF (green) and microglia (Iba-1, red) in the TNC in the VEH and NTG groups on day 11. Scale bar = 50 μm. **f** The white box was amplified. Scale bar = 20 μm. The white arrow indicates some BDNF proteins expressed in neurons

We investigated whether the expression of p38-MAPK, p-p38-MAPK and BDNF changed in the TNC following NTG injections. The Western blot results showed that the levels of p-p38-MAPK and BDNF were increased markedly. The increases in p-p38 and BDNF were detected on day 7 and lasted until day 11. However, the level of total p38-MAPK protein in the TNC was not different from that in VEH mice (Fig. 7a-d). Furthermore, double immunostaining showed that on day 11, BDNF was more abundantly expressed in microglia cells than that in control mice (Fig. 7e). However, we also found some BDNF expression in neurons, as shown by the arrow in Fig. 7f.

**Fig. 8:**
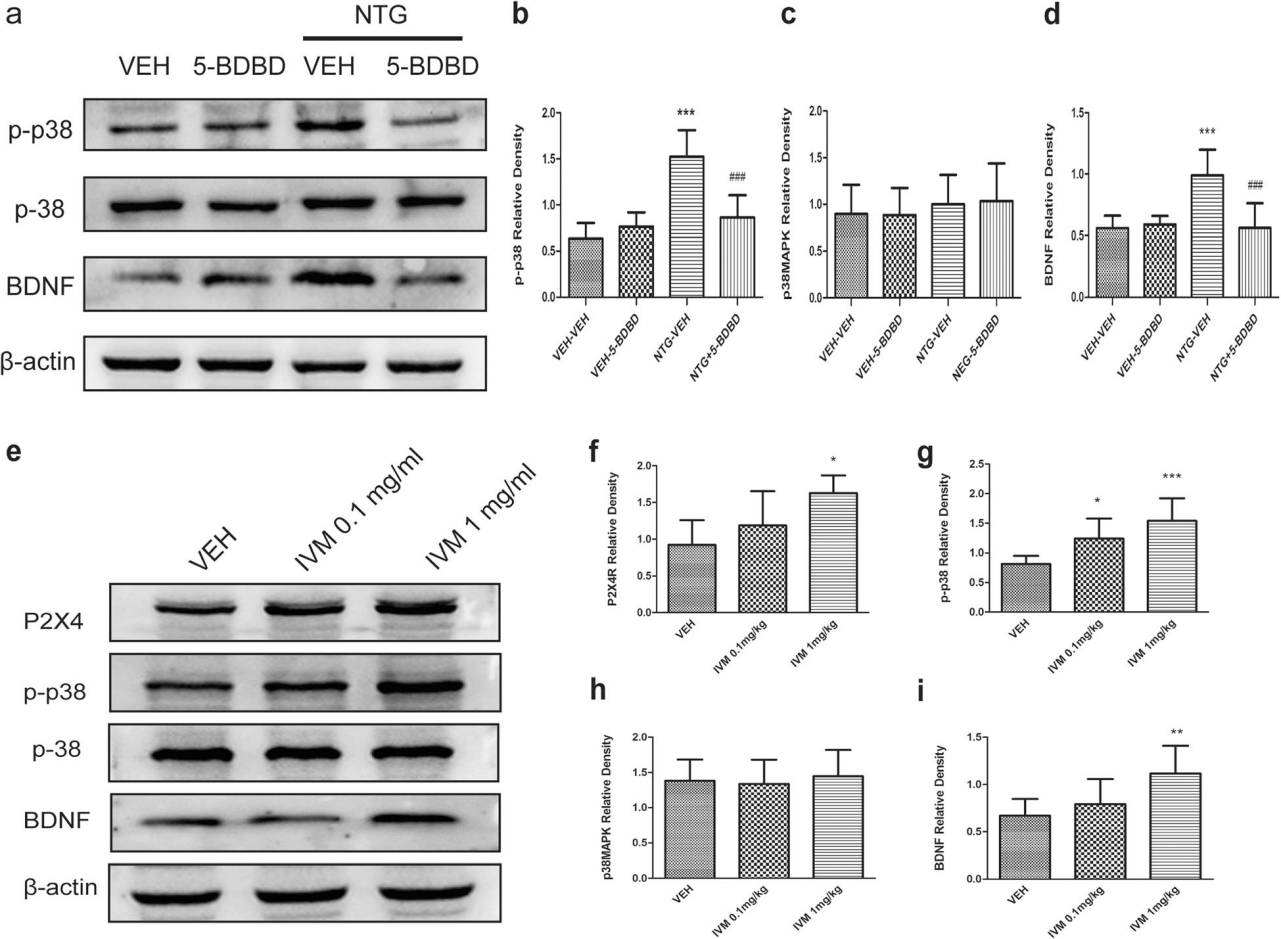
The levels of p38, p-p38-MAPK and BDNF after pharmacological inactivation or activation of P2X4Rs. **a** Representative images of Western blot results showing the expression of p38, p-p38 and BDNF proteins after 5-BDBD treatment. **b**-**d** The histogram presents the quantitative analysis of p-p38 (**b**), p38MAPK (**c**) and BDNF (**d**) in the TNC. *n* = 5/group. ***p < 0.001 versus VEH-VEH, ###*p* < 0.001 versus NTG-VEH. (**e**) Representative images of Western blot results showing the expression of P2X4, p38, p-p38 and BDNF proteins after IVM treatments. **f**-**i** The histogram presents the quantitative analysis of P2X4 (**f**), p38 (**g**), p-p38 (**h**) and BDNF (**i**) in the TNC. Data are presented as the mean ± SD. *n* = 6–8/group, One-way ANOVA and Tukey’s multiple comparison test. *p < 0.05, **p < 0.01, ***p < 0.001 versus VEH

With regard to the function of P2X4Rs in BDNF release or synthesis, we measured the levels of p38-MAPK, p-p38-MAPK and BDNF in the TNC after pharmacological inactivation or activation of P2X4Rs. The Western blotting results showed that p-p38-MAPK and BDNF were increased following NTG injection, and 5-BDBD treatment significantly reduced p-p38-MAPK and BDNF levels (Fig. 8a-d). In addition, p-p38-MAPK and BDNF were increased in the TNC after daily IVM injection compared to those after VEH injection. However, the level of p38-MAPK did not change (Fig. 8e-i).

### Chronic ANA-12 treatment attenuates hyperalgesia induced by chronic intermittent NTG

**Fig. 9:**
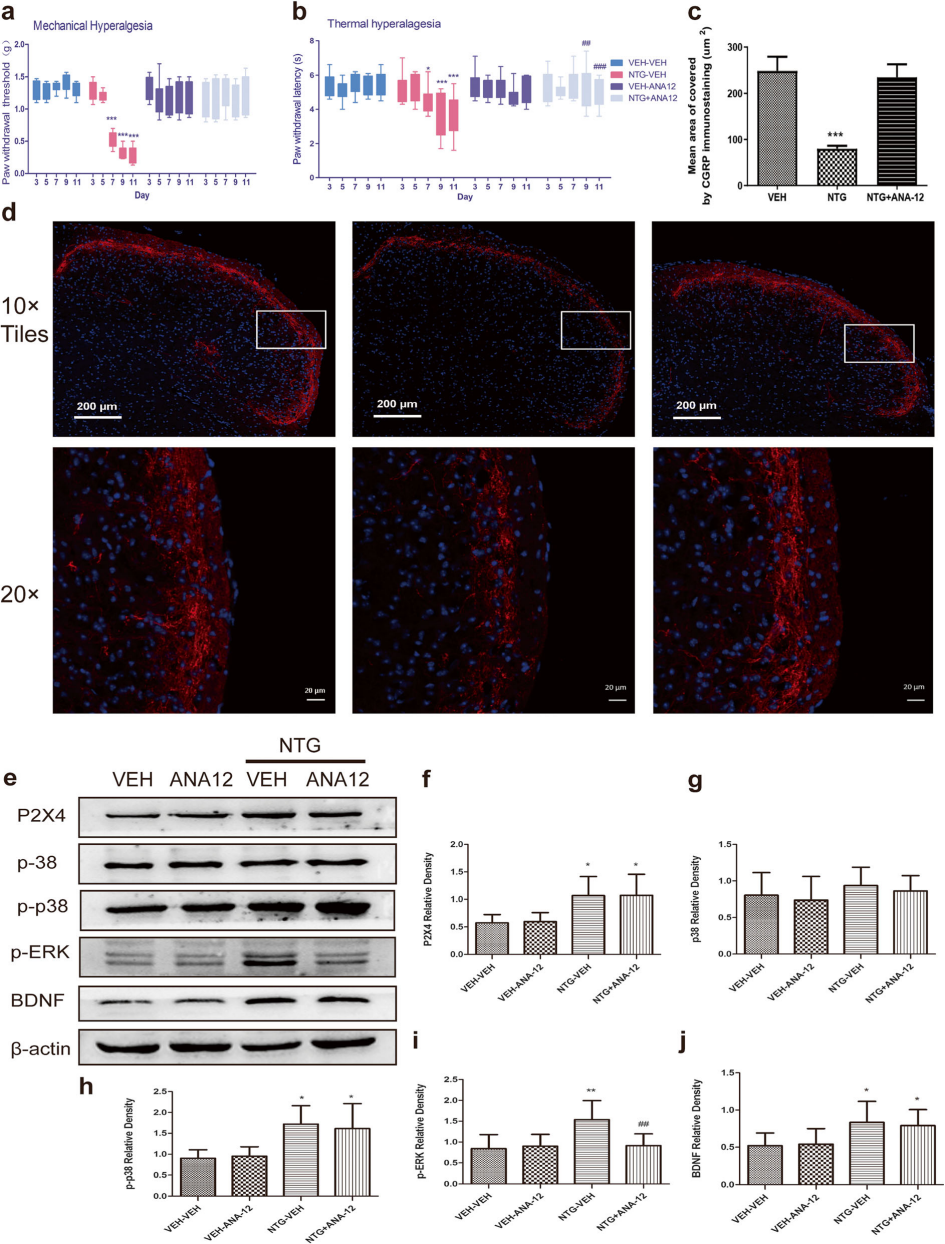
Effect of ANA-12 on NTG-induced migraine-related behavioural and neurochemical changes. Mice were treated daily with vehicle (VEH) or ANA-12 (1 mg/kg, i.p.) for 11 days. **a**, **b** Mechanical and thermal sensitivity were assessed in different groups. *p* < 0.01 for drug, time, and interaction. Two-way RM ANOVA and Bonferroni post hoc analysis. *n* = 10/group. *p < 0.01, ***p < 0.001 versus VEH-VEH group. ##*p* < 0.01, ###*p* < 0.001 versus NTG-VEH group. (**c**) Mean area covered by CGRP-immunoreactive fibres in different groups. *n* = 7/group, ***p < 0.001 versus VEH. **d** Representative photos of CGRP-immunoreactive staining in the TNC of the VEH, NTG and NTG + 5-BDBD groups under a 10 × objective lens (upper row, scale bar = 200 μm) or 20 × objective lens (lower row, scale bar = 20 μm). The white box was enlarged. **e** Representative Western blot results for P2X4, p38, p-p38, BDNF and p-ERK in the different groups. **f**-**j** The histogram presents the quantitative analysis of P2X4 (**f**), p38 (**g**), p-p38(**h**), p-ERK (**i**) and BDNF (**j**) in the TNC. *n* = 6–8/group. Data are presented as the mean ± SD. One-way ANOVA. *p < 0.05, **p < 0.01 versus VEH-VEH, ##*p* < 0.01 versus NTG-VEH

We demonstrated that P2X4Rs stimulation mediated BDNF release. We next determined whether BDNF was related to migraine-associated hyperalgesia following treatment with ANA-12, a TrkB receptor inhibitor. As mentioned earlier, chronic NTG injection produced marked mechanical (Fig. 9a) and thermal (Fig. 9b) hyperalgesia on days 7, 9, and 11 compared with vehicle injection. However, treatment with ANA-12 for 11 days significantly increased the PWT and PWL (Fig. 9a, b). This result indicated that BDNF may be responsible for the mechanical and thermal hyperalgesia induced by NTG.

### Chronic ANA-12 treatment attenuates CGRP release and ERK phosphorylation in the TNC

According to the immunofluorescence staining results, chronic NTG injection increased CGRP release in the TNC. However, ANA-12 treatment significantly reversed this change (Fig. 9c, d). Furthermore, the Western blot data showed that p-ERK was also increased with NTG injection compared to that with VEH injection, while ANA-12 significantly reduced the phosphorylation of ERK levels following daily treatment (Fig. 9e, i). In addition, ANA-12 had no effect on the expression of P2X4, p38-MAPK or p-p38-MAPK, which indicated that BDNF is downstream of P2X4R and p38-MAPK (Fig. 9e-j). Overall, our results indicate that P2X4/p38/BDNF signalling may contribute to recurrent NTG-induced central sensitization.

## Discussion

The present data in our study indicate that repeat NTG stimulation can induce mechanical and thermal hypersensitivity and an increase in p-ERK and CGRP release. The observed behavioural and neurochemical changes can be interpreted as a central sensitization phenomenon. Moreover, in these recurrent NTG injection mice, we found an enhancement of P2X4 and BDNF expression levels in the TNC. Negative P2X4-BDNF modulators can reverse hyperalgesia and the change in migraine biomarkers induced by repeated NTG injection. These data provide evidence that the TNC P2X4-BDNF pathway in microglia is a key signalling mechanism that regulates chronic migraine pathophysiology. BDNF is an important molecule mediating this microglia-neuron crosstalk.

### Animal model of chronic migraine

The pathophysiology of migraine chronification is not fully understood. Currently, a large number of CM studies focus on functional MRI or positron emission tomography (PET), while basic research is relatively slow, in part due to a lack of reliable animal models [21]. Current animal models of CM mainly include two types. One type is based on the repeated stimulation of pain-sensitive intracranial structures, such as repeated applications of inflammatory soup (IS) to the dura mater [22]. The other type is the systemic infusion of vasodilating agents, such as the repeated intraperitoneal administration of NTG. There are also some rare CM models, such as recurrent spreading depression or altering the endogenous pain modulating system [23].

In our study, we developed a mouse CM model with repeated NTG injections that was first described by Pradhan et al. [9]. In his research, chronic intermittent treatment with NTG not only produced acute mechanical hypersensitivity of the hind paw after each injection but also induced long-lasting basal hyperalgesia, which was alleviated by the migraine-preventive treatment of topiramate. This chronic basal hyperalgesia persisted for several days after the last NTG exposure. Interestingly, clinical studies have reported that migraine patients are more likely to show cephalic cutaneous hypersensitivity than extracephalic hypersensitivity [24]. Few studies measured the periorbital von Frey thresholds after repeated systemic NTG injections. Because of the difficulties in practice and unstable results, a decrease or no change in periorbital thresholds has been reported after NTG injection [25]. Therefore, in our study, we only measured hind paw mechanical hyperalgesia. However, extracephalic hyperalgesia (such as hindpaw) has been treated as the sign of sensitization of the third-order neurons (thalamus), not second-order neurons (TNC). Considering TNC has direct neuronal connections to thalamus, through this pathway, the continuous discharge of TNC neurons will certainly lead to sensitization of thalamic neurons. So, hindpaw hyperalgesia only provide an indirect evidence of TNC sensitization.

In addition to mechanical hyperalgesia, NTG can also induce thermal hypersensitivity of the hind paw [26]. We used an increasing-temperature hot plate apparatus to measure the PWL. In an acute NTG-induced migraine animal model, a decrease in the PWL was detected within 30 min and subsided 4 h after injection. In this study, we first explored the hind paw PWL in a chronic mouse model of migraine. Repeat injection of NTG produced marked thermal paw hyperalgesia on days 7, 9, and 11 compared with vehicle injection, which is consistent with the change in mechanical hyperalgesia. Similar effects were reported by Mahmoudi J et al., who showed that chronic NTG administration was able to evoke thermal hypersensitivity in Wistar rats [27]. Few studies have observed this thermal response in mice. Farajdokht F used the latency to tail withdrawal in a hot water bath (48 ± 0.5 °C) to determine thermal sensitivity and found that the repeated intermittent injection of NTG gradually produced thermal hyperalgesia [28]. This is the first time that we have used a hot plate apparatus to observe hind paw hyperalgesia in mice. In addition to evoking hind paw hyperalgesia, repeated NTG injections can also induce other migraine-related behaviours, such as head grooming behaviours, activity reduction, and light-aversive behaviours.

### CGRP and p-ERK are related to central sensitization in CM

A reliable animal model of CM should not only mimic the primary clinical phenotype of CM patients but also reflect changes in migraine-related biomarkers. CGRP is a widely used biomarker of migraine, and it is implicated in the pathology of migraine by promoting the development of peripheral and central sensitization. The synthesis of CGRP is completed in the cell bodies of TG neurons; then, CGRP is transported throughout the axon from the neuronal cell body to the peripheral and central terminals of the axon [29]. CGRP release in the meninges and TG initiates and sustains the peripheral sensitization of primary trigeminal neurons. CGRP release in the TNC causes activation or sensitization of second-order neurons and promotes the development of central sensitization. Many basic research and clinical studies have reported changes in CGRP concentrations in jugular venous blood and cerebrospinal fluid as well as the TG and TNC [30]. In our study, we found that CGRP immunostaining was striking in the superficial lamina of the TNC and presented a specific pattern of staining without distinct cellular localization, as it is presumed to be located in presynaptic afferent terminals. Our results show that NTG treatment reduced the area covered by CGRP immunostaining, which is consistent with our team’s previous studies. The reduced CGRP-innervated area may be related to an increased release in presynaptic afferent terminals. Greco and colleagues indicated that the NTG-induced reduction in CGRP lasted for 4 h [31]. Thus, CGRP gene transcription may increase in the TG, and newly synthesized CGRP may restore vesicle stores and may be transported throughout the axon to the TNC. Therefore, some studies found that CGRP levels in the TNC were elevated or did not change after NTG treatment [32, 33]. These differences were largely due to the different time points of testing.

Although c-Fos and p-ERK can be used as markers for neuronal activation following noxious stimulation or tissue injury, p-ERK is also a good marker for central sensitization [34]. The mechanisms underlying c-Fos-mediated central sensitization are largely unknown, although c-Fos has been widely used for many years as a pain marker. Accumulating evidence indicates that p-ERK, unlike c-Fos, induces and maintains central sensitization by increasing the activity of α-amino-3-hydroxy-5-methyl-4-isoxazolepropionic acid (AMPA) and N-methyl-D-aspartate (NMDA) receptors and suppressing the activity of potassium Kv4.2 channels. p-ERK can also activate the transcriptional factor cAMP response element-binding (CREB), which is critical for neuronal plasticity and the hyperexcitability of nociceptive neurons. NTG infusion significantly increased the level of p-ERK in the dura mater, TG, and TNC [35]. Isosorbide dinitrate (ISDN), as an NO donor similar to NTG, also significantly increased the number of p-ERK-immunoreactive cells in the medullary dorsal horn (MDH) [36], and p-ERK is closely correlated with pain behaviour and ongoing activity of trigeminal wide-dynamic range (WDR) neurons. In our study, we demonstrated that NTG can also induce an increase in p-ERK levels, which is consistent with the results of previous studies. Considering that non-noxious stimuli are also able to induce c-Fos in neurons, p-ERK appears to be a better marker than c-Fos for central sensitization.

### P2X4Rs in the release of BDNF

P2X4Rs are ATP-gated channels with high calcium permeability. Accumulating evidence suggests that P2X4Rs are expressed in central microglia and peripheral macrophages. In peripheral inflammatory responses, the activation of P2X4Rs evoked calcium influx and p38-MAPK phosphorylation, resulting in the release of prostaglandin E2 (PGE2) [37]. However, in microglia, P2X4Rs mainly cause the release of BDNF, which is a key molecule for maintaining pain hypersensitivity after nerve injury. In this study, we found that stimulating P2X4Rs increased BDNF expression and caused BDNF release. The synthesis of BDNF was increased starting at 60 min and peaked at 120 min, which was consistent with the result of BDNF release. Salter et al. reported that the peak phase of BDNF synthesis and release was 60 min after ATP stimulation [38]. This discrepancy is mainly due to the different cell types (BV2 cells vs. primary microglia). Previously, due to the lack of P2X4R-specific inhibitors, TNP-ATP (an antagonist of P2X1-4R) and PPADS (an antagonist of P2X1–3,5,7R but not P2X4Rs) were used to examine the role of P2X4Rs in BDNF release and synthesis. These authors found that TNP-ATP prevented the ATP-evoked increase in BDNF release and synthesis, but PPADS had no effect. In our study, we used 5-BDBD, a P2X4R-specific inhibitor, and found that pre-incubation with 5-BDBD, ATP had no effect on the level of BDNF in the cell lysates or supernatant. Together, the results indicate that P2X4Rs are sufficient to mediate the BDNF release and synthesis evoked by ATP. Among the P2X family, P2X4 demonstrates the highest Ca2+ permeability. The Ca^2+^-dependent activation of p38-MAPK has been demonstrated in a number of cell types [38]. Therefore, we speculated that p38-MAPK is the key intracellular signalling pathway through which the stimulation of P2X4Rs leads to the release and synthesis of BDNF. Our results demonstrate that in BV2 cells, ATP increased p-p38-MAPK expression in cell lysates, and inhibiting p38-MAPK blocked the release of BDNF. Thus, it is possible that p38-MAPK activity may contribute to the ATP-evoked release of BDNF.

### P2X4R-BDNF pathway in chronic migraine

ATP is a well-known allogenic substance that activates purinergic receptors (P2X and P2Y receptors). The source of ATP may be endothelial cells, aggregating platelets, neurons, microglia, or even astrocytes [39]. The role of the purinergic signalling system in the pathophysiology of migraine was proposed more than 30 years ago. Since then, different subtypes of P2 receptors have been elucidated, including the P2X3R, P2X7R, P2Y1R, and P2Y2R [40–42]. Among them, the P2X3R is the most widely studied. A number of years after the discovery of the P2X4R, several studies demonstrated that P2X4Rs are necessary and sufficient for pain hypersensitivity using diverse animal models of neuropathic pain. Very little is known, however, about the role of P2X4R in the pathogenesis of migraine.

Previous results from our group indicated that the expression of microglia P2X4Rs increased in the TNC in animal models of chronic migraine. NTG-induced pain behaviours, as well as migraine-related neurochemical signalling, are reversed by blocking P2X4Rs, which is consistent with the results of this study. However, recent evidence has been inconsistent in supporting the role of P2X4Rs in pain hypersensitivity. Mapplebeck and colleagues proposed that after peripheral nerve injury, females do not upregulate P2X4Rs and use a microglia-independent pathway to mediate pain hypersensitivity [43]. They proposed that adaptive immune cells, possibly T cells, may mediate pain hypersensitivity in female mice. Therefore, the prevalence of migraine demonstrates sex differences. The reason may be that the role of microglia in pain is sexually dimorphic. Although female sex is not a risk factor associated with migraine progression [2], one limitation of the present study is that we only used male mice. In future studies, female animals should be included to explore the sex differences in the pathogenesis of migraine.

To evoke central sensitization, the P2X4Rs in microglia must initiate a process that is communicated to neurons in the TNC. Some studies have introduced BDNF as a pivotal mediator for microglia–neuron communication. A series of studies showed that the activation of microglia P2X4Rs stimulated the synthesis and release of BDNF, and BDNF then acted on its high affinity receptor, TrkB. The activation of TrkB in dorsal horn neurons regulates neuron activity, which contributes to reduced inhibition and increased excitation [44]. Thus, P2X4R-stimulated microglia release BDNF as the core signalling pathway for microglia–neuron communication in the pathogenesis of neuropathic pain.

In this study, we found that stimulating P2X4Rs with ATP promoted the synthesis and release of BDNF by BV2 cells. In addition, the level of BDNF was increased markedly in the TNC following NTG injection. Immunostaining showed that BDNF was abundantly expressed in microglia cells, but low expression was observed in TNC neurons. It is generally known that the expression of BDNF in neurons also plays a critical role in the development of the nervous system. However, in nerve injury models of neuropathic pain, nociceptive neuron-BDNF null mice do not develop pain-like behaviour, suggesting no major role of BDNF derived from small sensory-neurons under neuropathic conditions [45].

To determine whether BDNF was related to migraine-associated hyperalgesia, we used ANA-12, a TrkB receptor inhibitor, in NTG mice. Chronic treatment with ANA-12 for 11 days significantly increased the PWT and PWL, and ANA-12 attenuated CGRP release and ERK phosphorylation in the TNC, indicating a pro-nociceptive role of BDNF. However, the use of the TrkB inhibitor ANA-12 did not provide a solid answer because neurotrophin 4/5 (NT4/5), which also belongs to the neurotrophin family of trophic factors, can also activate the TrkB receptor. Martins et al. indicated that chronic and episodic migraine patients showed higher NT4/5 levels than control individuals [46]. Moreover, NT4-mediated TrkB activation regulates morphine-induced analgesia [47]. Therefore, using the genetic depletion of BDNF from microglia or neuron animals may provide a good model to evaluate the role of BDNF in migraine or other animal models of pain.

## Conclusions

Our findings indicate that the P2X4Rs may contribute to central sensitization after repeated NTG stimulation. Pharmacological blockade of P2X4Rs evidently prevents migraine-associated hyperalgesia, CGRP release and ERK phosphorylation. We suggest that the effectiveness of P2X4Rs in migraine progression may be due to the promotion of the synthesis and release of BDNF in microglia. BDNF is a key molecule mediating microglia-neuron signalling. Therefore, the modulation of P2X4R-BDNF signalling function may be a novel therapeutic avenue for the treatment of chronic migraine.

## Data Availability

The data used in this article are available if necessary.

## Abbreviations

AMPA: α-amino-3-hydroxy-5-methyl-4-isoxazolepropionic acid
ATP: Adenosine triphosphate
BDNF: Brain-derived neurotrophic factor
CGRP: Calcitonin gene related peptide
CM: Chronic migraine
CREB: cAMP response element-binding
CSD: Cortical spreading depression
IITC: Increasing-temperature hot plate apparatus
IS: Inflammatory soup
ISDN: Isosorbide dinitrate
MDH: Medullary dorsal horn
NMDA: N-methyl-D-aspartate
NTG: Nitroglycerin
OD: Optical density
P2X4R: P2X4-receptor
p38MAPK: phosphorylated p38 mitogen-activated protein kinase
p-ERK: phosphorylated extracellular regulated protein kinases
PET: Positron emission tomography
PGE2: Prostaglandin E2
PWL: Paw withdrawal latency
PWT: Paw withdrawal threshold
ROS: Reactive oxygen species
TG: Trigeminal ganglion
TNC: Trigeminal nucleus caudalis
TrkB: Tropomyosin-related kinase B
VEH: Vehicle
WDR: Wide-dynamic range
